# Integrated quantitative sensory testing and psychological phenotyping dataset for capsaicin-induced neuropathic pain susceptibility

**DOI:** 10.1016/j.dib.2026.113141

**Published:** 2026-08-06

**Authors:** Jörn Lötsch, Violeta Dimova

**Affiliations:** a Goethe University, Institute of Clinical Pharmacology, Faculty of Medicine, Theodor-Stern-Kai 7, 60590 Frankfurt am Main, Germany; b University of Helsinki, Faculty of Medicine, Haartmaninkatu 8, P.O. Box 63, 00014 University of Helsinki, Finland; c Fraunhofer Institute for Translational Medicine and Pharmacology (ITMP), Theodor-Stern-Kai 7, 60596 Frankfurt am Main, Germany

**Keywords:** Psychophysics, Human, Pain research, Experimental pain

## Abstract

Experimental pain models are used to investigate the analgesic effects in healthy volunteers during the early stages of drug development. However, the predictive value of these models for clinical pain requires continuous reinforcement. One question is whether neuropathic pain patterns can be induced in healthy individuals. Another question is whether healthy volunteers can be pre-selected based on the inducibility of neuropathic-like sensory patterns under controlled experimental conditions. A third question is whether psychological traits contribute to this susceptibility. The present dataset originates from a study that addressed these questions by combining standardized sensory and psychological phenotyping of the same participants. The working hypothesis was that patterns of clinical neuropathic pain could be induced in a subgroup of healthy volunteers by applying topical capsaicin and that susceptible individuals could be identified based on their psychological phenotype.

To evaluate neuropathic pain inducibility, a standardized quantitative sensory testing (QST) battery, developed by the German Research Network on Neuropathic Pain, was administered to 110 healthy volunteers (46 men aged 18–36 years) at two sites: the untreated contralateral skin and the contralateral skin sensitized with topical capsaicin. The QST battery includes 11 parameters that cover thermal and mechanical detection and pain thresholds, mechanical pain sensitivity, and temporal pain summation (wind-up ratio), as well as vibration and pressure pain thresholds. Z-scores were computed against a published normative reference cohort matched by age and sex, enabling direct comparison of individual sensory profiles with those of patients with clinical neuropathic pain. Additionally, seven validated psychometric questionnaires covering dispositional optimism, depressive mood, somatoform symptoms, state anxiety, pain catastrophizing, pain anxiety, and pain vigilance were administered to all participants.

The dataset is provided as three comma-separated values (CSV) files. The first file contains z-transformed QST data in an 110 × 23 matrix (11 QST parameters at two sites per participant plus unique identifier). The second file contains psychometric questionnaire total scores in an 110 × 8 matrix (7 psychological parameters plus unique identifier). The third file contains participant metadata, including age, sex, test and control site coding for QST, and a binary classification variable indicating whether the QST profile at the capsaicin-treated site was consistent with a neuropathic sensory pattern. All three files have consistent subject identifiers.

This dataset can serve as a resource for future human pain research investigations. Integrating standardized sensory phenotyping and comprehensive psychological characterization of the same individuals allows for the systematic study of psychological predictors of pain sensitivity and neuropathic pain susceptibility. Precomputed neuropathic pattern classifications facilitate supervised machine learning approaches, and z-transformed QST values enable direct benchmarking against established clinical neuropathic pain profiles. The dataset also supports secondary analyses, such as investigating sex differences in experimental pain sensitivity. Additionally, it can serve as an independent reference cohort for machine learning applications. All data are fully anonymized and structured.

Specifications Table

**Table:** 

Subject	Health Sciences, Medical Sciences & Pharmacology
Specific subject area	Quantitative sensory testing and experimental pain model data in healthy adults, with and without capsaicin sensitization.
Type of data	Table, Raw, Processed, Comma-separated values (csv)
Data collection	Healthy adults (no chronic pain) underwent standardized quantitative sensory testing (QST) following the German Research Network on Neuropathic Pain protocol, on untreated and capsaicin-sensitized skin. Instruments: Medoc TSA-II (thermal), JTECH Commander algometer (pressure), and calibrated von Frey filaments and pinprick stimulators (mechanical). Seven validated questionnaires assessed pain catastrophizing, pain anxiety, pain vigilance, depression, state anxiety, optimism, and somatoform symptoms. Exclusion: recent drug intake, pain conditions, or current disease. QST values were log-transformed and z-standardized against age-, sex-, and site-matched reference values.
Data source location	Goethe - University, Frankfurt am Main, Germany
Data accessibility	Repository name: Mendeley Data Data identification number: doi: 10.17,632/x7d986jg75.1 Direct URL to data: https://data.mendeley.com/datasets/x7d986jg75/1 Instructions for accessing these data: Dataset name in Mendeley data: “Integrated quantitative sensory testing and psychological phenotyping dataset for capsaicin-induced neuropathic pain susceptibility”. Data can be downloaded as three comma-separated values (CSV) files.
Related research article	Lötsch J, Dimova V, Hermens H, Zimmermann M, Geisslinger G, Oertel BG, Ultsch A. Pattern of neuropathic pain induced by topical capsaicin application in healthy subjects. Pain. 2015;156(3):405–14.

## 1 Value of the Data

• **Multimodal dataset linking sensory and psychological pain phenotypes:** This dataset combines standardized quantitative sensory testing (QST) data with a set of psychological questionnaire scores in the same 110 healthy participants, enabling joint analyses of sensory and psychological dimensions of pain sensitivity. The linkage between the three files via consistent subject identifiers facilitates cross-domain analyses that are rarely available in a single openly accessible dataset.
• **Experimental neuropathic pain model with clinical QST validation:** QST was performed subsequently under two conditions (untreated skin and topical capsaicin-sensitized skin) using the standardized DFNS clinical battery. Z-scores were computed against a published normative reference cohort, enabling direct comparison with clinical neuropathic pain profiles and making the dataset suitable for benchmarking experimental pain models against clinical pain conditions.
• **Pre-classified neuropathic pain inducibility:** Each participant carries a binary classification, identifying the subgroup (18% of participants) in whom topical capsaicin induced a QST profile resembling clinical neuropathic pain in 50–60% of parameters. This label enables supervised machine learning analyses aimed at predicting neuropathic pain inducibility from sensory or psychological baseline characteristics.
• **Psychological phenotyping for pain research:** Seven validated psychometric instruments assessing dispositional optimism, depressive mood, somatoform symptoms, state anxiety, catastrophizing, pain anxiety, and pain vigilance were administered to all participants. These psychological factors were shown explaining individual differences in pain sensitivity, consistent with the original finding that pessimistic life orientation predicted neuropathic pain inducibility.
• **Canonical dataset for machine learning in pain research:** The dataset's structure, including standardized, z-transformed continuous variables, binary classification labels, and matched psychological scores, makes it suitable for developing and testing statistical and computational methods in human pain research. It may serve as a validation dataset for machine-learning-based analyses of quantitative sensory studies, including feature selection, clustering, and classification benchmarking.
• **Support for research on sex differences and individual variability in pain:** With 46 men and 64 women aged 18–36 years, the dataset is well-suited for studying sex differences in experimental pain sensitivity and neuropathic pain inducibility. The availability of both sensory and psychological measures in the same individuals additionally supports research on the interplay between demographic and pain-related psychological factors.

## 2 Background

The dataset was collected as part of a project to develop predictive human experimental pain models.

Quantitative sensory testing (QST) was performed on healthy volunteers with and without topical capsaicin sensitization [1]. The goal was to elicit neuropathic pain-like sensory patterns to support the development and optimization of pain therapies using human experimental pain models. In addition, it was explored whether healthy subjects, in whom a pattern of neuropathic pain could be induced by topical capsaicin application, differed from the other healthy participants with respect to their psychological phenotype [2].

For clinically relevant patterns of neuropathic pain, a standardized QST battery [3,4] was used to record sensory profiles from untreated and capsaicin-sensitized skin. While most QST values from untreated skin matched reference ranges, capsaicin produced deviations in several parameters; some subjects showed substantial resemblance to neuropathic profiles. This offers the opportunity enrolling healthy subjects in early-phase clinical studies of treatments aimed at neuropathic pain. The preselection of suitable subjects could be guided by psychological properties, as a more pessimistic life orientation was associated with experimental inducibility of a neuropathy-like pain pattern in 18% of the study participants [2].

This dataset differs from the previously published dataset in two ways. First, the psychological questionnaire scores ("psy_questionnaire_scores.csv") are unique to this dataset and were not included in [5]. Second, z-transformed QST values are provided for both the capsaicin-treated test site and the untreated contralateral control site. The previously published dataset only contains control site values. The dataset provides a canonical resource for machine-learning approaches to pain research [6] and can support the refinement of experimental pain models toward better reflection of clinical neuropathic pain.

## 3 Data Description

The dataset includes three comma-separated values (CSV) files, each playing a specific role in representing pain or psychological data and the associated metadata. All files maintain consistent row order by subject ID, facilitating cross-referencing.

### 3.1 qst_z_scores.csv

This file contains z-transformed QST data in a 110 × 23 matrix. The first column ("ID") lists arbitrary subject identifiers, consistent across all three dataset files. The remaining columns contain z-scores for 11 QST parameters measured at the untreated contralateral control site (z_*_contr) and the capsaicin-treated test site (z_*_test) for each participant, where the asterisks stand for the parameter abbreviations listed in the "Variable" column of Table 1. Z-transformation was performed against an age- and sex-matched published reference cohort, standardizing for site, sex, and age, according to $z_{QST,individual}=\frac{QST_{individual}-QST_{reference}}{Standard\;deviation_{reference}}$, where $QST_{reference}$ and $Standard\;deviation_{reference}$ are the published reference values and standard deviations matched for the subject's sex, age, and tested body site (specific reference means and standard deviations are provided in Table S1 of [7]). The signs of z-scores were adjusted so that z > 0 indicates increased sensitivity and z < 0 indicates decreased sensitivity.

**Table 1:** Eleven components of the quantitative sensory testing battery of the German Research Network on Neuropathic Pain. Table adapted from [1] and similar to [5]. The values provided in the files were obtained after the basic data processing described in the fourth column of the table.

	Test	Variable	Sensory dimension	Basic data processing	Original Unit
Thermal testing	**CDT**	Cold detection threshold	Topical application of cold stimuli, baseline t° = 32 °C, decreasing by 1 °C/s	Difference from baseline 32 °C of the mean of 3 measurement repetitions, log*-transformation*	°C-32
	**WDT**	Warm detection threshold	Topical application of warm stimuli, baseline t° = 32 °C, increasing by +1 °C/s	Difference from baseline 32 °C of the mean of 3 measurement repetitions, log*-transformation*	°C-32
	**TSL**	Thermal sensory limen	Topical application of alternating cold and warm stimuli	Difference in the means of the 3 warmth and the 3 cold detection thresholds, log-transformation	Δ °C
	**CPT**	Cold pain threshold	Topical application of cold stimuli, baseline t° = 32 °C; decreasing by 1 °C/s	Mean of the 3 measurement repetitions	°C
	**HPT**	Heat pain threshold	Topical application of warm stimuli, baseline t° = 32 °C, increasing by 1 °C/s	Mean of the 3 measurement repetitions	°C
Pressure pain threshold	**PPT**	Pressure pain threshold	Application of blunt pressure stimuli to *M. thenar* for the hand area and *M. abductor hallucis* for the foot area	Mean of the 3 measurement repetitions, log-transformation	kPa
Mechanical pain threshold	**MPT**	Mechanical pain threshold	Application of pinprick stimuli (8 to 512 mN; contact area 0.2 mm) following staircase paradigm, starting force of 8 mN	Geometric mean of the 5 ascending and 5 descending stimuli, log-transformation	mN
Stimulus-response function	**MPS**	Mechanical pain sensitivity for pinprick stimuli	Application of pinprick stimuli and tactile stimuli in a balanced order, 0 - 100 pain rating scale.	Geometric mean of the pain ratings of the 35 pinprick stimuli, log-transformation	rating
Wind-up	**WUR**	Wind-up ratio	Temporal summation of pinprick stimuli, application of single pinprick stimulus (256 mN) followed by train of 10 pinprick stimuli (256 mN, 1/s repetition rate), 0 - 100 pain rating scale	Ratio of the mean pain ratings of the 5 series of stimuli and the mean pain ratings of the 5 single stimuli, log-transformation	unitless
Mechanical detection threshold	**MDT**	Mechanical detection threshold	Application of von Frey hairs (forces 0.25 to 512 mN, diameter 0.5 mm), staircase paradigm, starting force of 16 mN	Geometric mean of the 5 ascending and 5 descending stimuli, log-transformation	mN
Vibration detection threshold	**VDT**	Vibration detection threshold	Application of descending vibration stimuli (64 Hz, 8/8 scale) to the *Processus styloideus radii* for hand area and *Malleolus medialis* for foot area	Mean of the 3 measurement repetitions	scale units (0–8)

In this dataset, the z-scores constitute the primary research data, as QST is routinely interpreted in z-transformed version within the DFNS framework and clinically relevant deviations from normal are defined on this standardized scale rather than in raw units [4]. Raw QST values are not provided separately; however, they can be recovered by reverse application of the z-standardization equation above, using the reference values from Table S1 of [7].

**Fig. 1:**
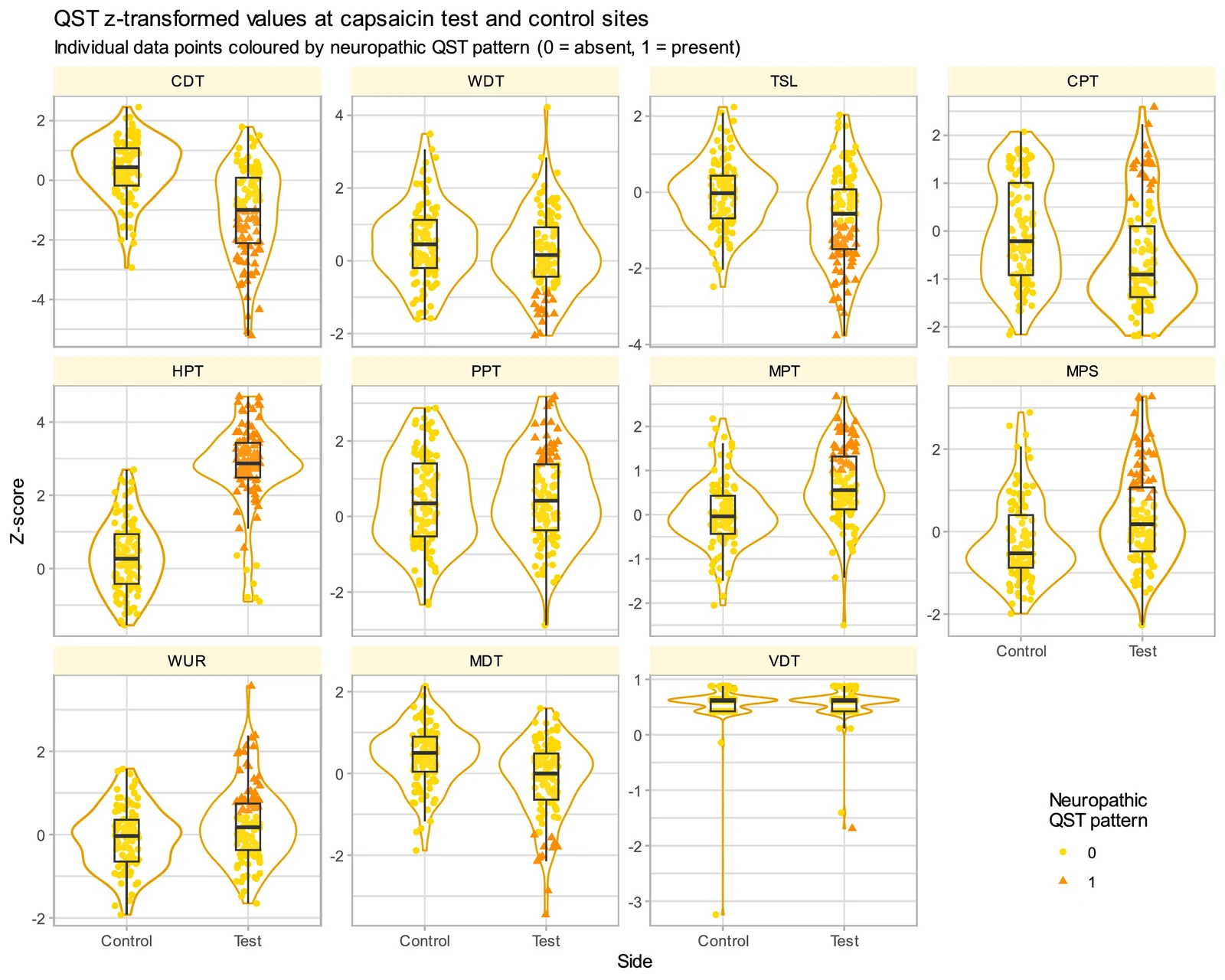
Distribution of QST parameters after z-transformation at the capsaicin-treated test site and the untreated contralateral control site in 110 healthy subjects. Violin plots display the full distribution of z-scores; overlaid box plots show the median and interquartile range. Individual data points are superimposed. Z-transformation was performed against an age- and sex-matched reference cohort, eliminating differences attributable to test site, sex, and age. Color and shape of data points indicate whether the individual QST profile at the test site was consistent with a neuropathic sensory pattern (orange, 1) or not (gold, 0). Z-scores at control sites cluster near zero, confirming correspondence with published reference values as expected in healthy subjects. CDT, cold detection threshold; CPT, cold pain threshold; HPT, heat pain threshold; MDT, mechanical detection threshold; MPS, mechanical pain sensitivity; MPT, mechanical pain threshold; PPT, pressure pain threshold; TSL, thermal sensory limen; VDT, vibration detection threshold; WDT, warm detection threshold; WUR, wind-up ratio. The figure displays the same data that are plotted in Fig. 1 in [1], and the color-coding of the neuropathy-like pattern is the same as in Fig. 2 in [1].

Full details of each parameter, including the sensory dimension assessed, measurement procedure, and basic data processing steps applied prior to z-transformation, are provided in Table 1. The resulting z-score distributions at both sites are illustrated in Fig. 1. It shows that z-scores at control sites cluster near zero as expected in healthy subjects, while deviations at the capsaicin-treated test site indicate experimentally induced sensitization in a subset of participants.

Subject IDs are consistent with those in the previously published dataset [5], enabling unambiguous matching of overlapping participants across the two datasets. The present dataset partially overlaps with a previously published dataset from the same research project. Of the 110 participants included here, 102 (92.7%) are also represented in that larger dataset.

### 3.2 psy_questionnaire_scores.csv

**Fig. 2:**
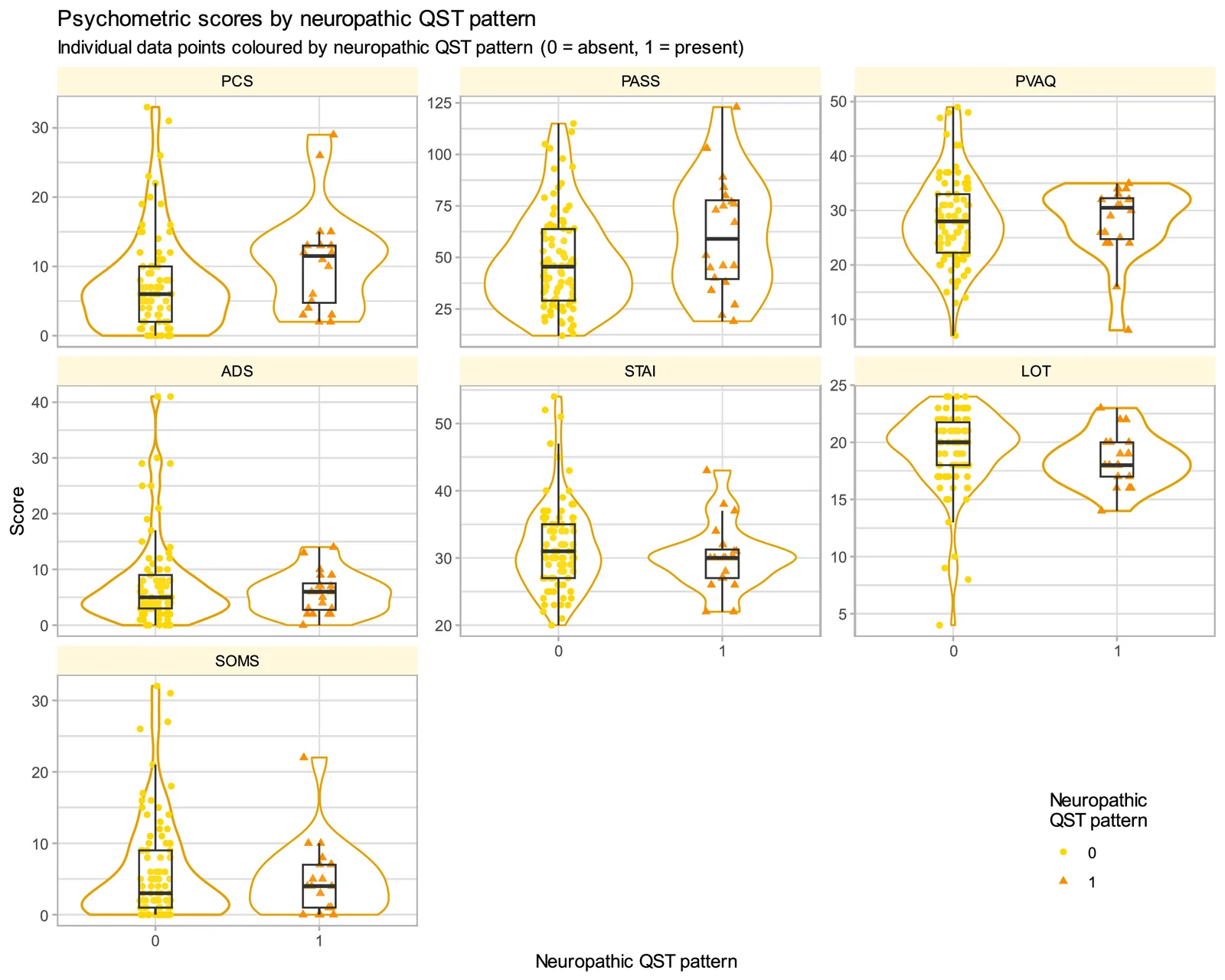
Psychometric questionnaire scores in 110 healthy subjects, grouped by neuropathic QST pattern. Violin plots display the full distribution of each score; overlaid box plots show the median and interquartile range. Individual data points are superimposed. Color and shape of data points indicate whether the individual QST profile at the capsaicin-treated test site was consistent with a neuropathic sensory pattern (orange, 1) or not (gold, 0). ADS, general depression scale; LOT, Life Orientation Test (optimism); PASS, Pain Anxiety Symptoms Scale; PCS, Pain Catastrophizing Scale; PVAQ, Pain Vigilance and Awareness Questionnaire; SOMS, Screening for Somatoform Disorders; STAI, State-Trait Anxiety Inventory. The figure displays the same data that are plotted in Fig. 1 in [2].

This file contains the same 110 cases in a 110 × 8 matrix of total scores for seven psychometric questionnaires administered to each participant, identified by the same arbitrary subject identifiers as in the first file. The instruments used include PCS (Pain Catastrophizing Scale), PASS (Pain Anxiety Symptoms Scale), PVAQ (Pain Vigilance and Awareness Questionnaire), ADS (“Allgemeine Depressionsskala”, German Version of Center for Epidemiologic Studies Depression Scale (CES-D)), STAI (State-Trait Anxiety Inventory), LOT (Life Orientation Test (optimism)), and SOMS (Screening for Somatoform Disorders). The scoring procedure and score range for each instrument are detailed in Table 2. Fig. 2 shows the distribution of questionnaire scores grouped by neuropathic QST pattern, illustrating that subjects in whom a neuropathy-like QST pattern was inducible tended toward more pessimistic attitude), consistent with the main finding of the original study [2].

**Table 2:** Seven psychological questionnaires included in the dataset. The values provided in the files represent the overall sum score of each questionnaire as described in the fourth column.

Questionnaire	Variable	Psychological dimension	Scoring	Score range
Pain Catastrophizing Scale	**PCS**	Pain-related catastrophizing (rumination, magnification, helplessness)	Sum of 13 items, each rated on a 5-point scale	0–52
Pain Anxiety Symptom Scale	**PASS**	Pain-related anxiety across cognitive, behavioral, and physiological domains (cognitive anxiety, avoidance, fearful appraisal, physiological anxiety symptoms)	Sum of items rated on a 6-point scale	0–240
Pain Vigilance and Awareness Questionnaire	**PVAQ**	Pain vigilance, awareness, preoccupation, and observation of pain	Sum of 16 items, each rated on a 6-point scale	0–80
Center for Epidemiologic Studies Depression Scale	**ADS (CES-D)**	Emotional, somatic, and cognitive symptoms of depressive mood in the preceding week	Sum of 20 items rated on a 4-point Likert scale	0–60
State-Trait Anxiety Inventory, state subscale	**STAI (STAI-X1)**	State anxiety comprising subjective feelings of apprehension, tension, and worry	Sum of 20 items rated on a 5-point scale	0–80
Life Orientation Test, revised	**LOT (LOT-R)**	Dispositional optimism: general expectancy of positive or negative outcomes across life situations	Sum of 6 scored items (3 positive, 3 negative; 4 filler items excluded), each rated on a 5-point scale	0–24
Screening for Somatoform Symptoms	**SOMS**	Somatoform symptoms: physical symptoms not explicable by organic causes, rated during the last seven days	Sum of 53 items rated on a 5-point Likert scale	0–212

### 3.3 participant_metadata.csv

The metadata file contains 110 rows and six columns. The first column lists subject IDs consistent with those in the other two files, enabling straightforward cross-referencing across all three files. Subject IDs are arbitrary numerical identifiers assigned during data management and do not follow a sequential or meaningful numbering scheme. They are consistent across all three files and with the previously published overlapping dataset [5], but carry no personal or demographic information and cannot be used to re-identify participants. The dataset is fully anonymized.

The remaining columns contain demographic and study characteristics:

• age_years: participant age in years
• sex_male_1: biological sex (1 = male, 0 = female)
• qst_test_site_code: coded body location of the capsaicin application (1 = hand, 2 = foot, 3 = forearm, 4 = face, 5 = back/shoulder/neck, 6 = back/pelvis, 7 = thigh)
• qst_control_site_code: coded body location of the contralateral control site, coded as above
• neuropathic_pattern: A binary variable derived from indicating whether the individual QST profile at the test site was consistent with a neuropathic sensory pattern (1 = present, 0 = absent).

## 4 Experimental Design, Materials and Methods

### 4.1 Participants and study design

Healthy volunteers of Caucasian ethnicity by self-assignment (n = 110, 46 men) were included. They were aged between 18 and 36 years (mean ± standard deviation: 25.1 ± 3.2 years). Inclusion criteria were age ≥ 18 years and no relevant current medical history. Exclusion criteria were drug intake during the previous week (except oral contraceptives and vitamin or hormone substituting drugs such as l-thyroxin), a current clinical condition involving pain, and current diseases according to questioning and medical examination. Before the experimental tests, all subjects completed training sessions with pain tests applied to an area different from the planned test and control areas. A trained investigator performed all measurements in accordance with the published instructions [3,4].

### 4.2 Standardized quantitative sensory testing (QST) battery

A standardized clinical QST battery established by the German Research Network on Neuropathic Pain (DFNS) [3,4] was used to assess pain sensitivity. The battery encompasses 11 tests yielding 13 parameters, administered in a fixed order. QST was performed twice in each subject: once on untreated limb skin (the "control" site) and again contralaterally on the capsaicin-hypersensitized area (the "test" site). The body area to be tested was randomly assigned; possible sites were the dorsal hand in the dermatome of *N. radialis* or the dorsal foot in the dermatome of *N. fibularis profundus*. Experimental hyperalgesia was induced at the test site by applying 150 mg capsaicin cream (0.2%, manufactured by the local Hospital Pharmacy) onto a 3 × 3 cm² skin area covered with a plaster for 30 min prior to testing [8]. Room temperature was maintained at 20–25 °C throughout. All measurements were taken by trained investigators in full adherence to the published protocol [3,4]. Full details of the sensory dimension assessed, measurement procedure, and basic data processing applied prior to z-transformation for each parameter are provided in Table 1, and distributions of QST Z-scores grouped side and marked for neuropathic QST pattern are shown in Fig. 1.

### 4.3 Thermal detection and pain thresholds

Cold detection threshold (CDT), warm detection threshold (WDT), thermal sensory limen (TSL), cold pain threshold (CPT), and heat pain threshold (HPT) were measured using a computer-controlled thermode (baseline 32 °C; ramp 1 °C/s; cutoffs 0–5 °C for cold, 50 °C for heat). Subjects pressed a button at the first perception of a temperature change or pain. Thresholds were averaged over three trials. TSL was assessed using alternating warm and cold stimuli. Subjects indicated the direction of change and any paradoxical heat sensations (PHS).

### 4.4 Mechanical detection and pain thresholds

Mechanical detection threshold (MDT) was assessed using von Frey filaments. These filaments had a range of 0.25–512 mN. Mechanical pain threshold (MPT) was assessed using calibrated weighted pinprick stimulators. These stimulators had a range of 8–512 mN. Thresholds were determined using the method of limits and calculated as the geometric mean of sub- and suprathreshold intensities.

### 4.5 Mechanical pain sensitivity (MPS)

Mechanical pain sensitivity (MPS) was assessed by applying pinprick stimuli of varying intensities five times, and subjects rated the pain on a scale from 0 to 100. Then, the geometric mean of the pain ratings was calculated.

### 4.6 Wind-up ratio (WUR)

Temporal summation of pain was evaluated by comparing pain ratings for a single 256-mN pinprick stimulus versus a series of ten repetitive stimuli (1 Hz). The wind-up ratio (WUR) was calculated by dividing the mean pain rating for the series by the mean rating for the single stimulus.

### 4.7 Vibration detection threshold (VDT)

The vibration detection threshold (VDT) was measured by applying a 64-Hz tuning fork to a bony prominence (*Processus styloideus radii* for the hand, or *Malleolus medialis* for the foot). Subjects indicated when the vibration was no longer perceptible, and the mean of three trials was recorded.

### 4.8 Pressure pain threshold (PPT)

Pressure pain threshold (PPT) was determined using a pressure algometer, which was applied at a constant rate until the subject reported the first sensation of pain, at *Musculus thenar* for the hand and *M. abductor hallucis* for the foot. The mean of three measurements was used.

### 4.9 Assessment of psychological factors

Seven psychometric questionnaires were administered to characterize participants' psychological profiles, grouped into three domains as follows.

### 4.10 Dispositional optimism

Dispositional optimism was assessed using the revised Life Orientation Test (LOT-R [9]; German version [10]), a 10-item instrument (three positive items, three negative items, four filler items) rated on a five-point scale (1 = strongly agree to 5 = strongly disagree). The sum of the six scored items yields a continuously rated optimism level (score range 0–24); higher scores indicate greater optimism.

### 4.11 General psychological factors

Depressive mood during the preceding week was measured with the Center for Epidemiologic Studies Depression Scale (CES-D, German version ADS [11]), a 20-item questionnaire rated on a four-point Likert scale (total score 0–60). Somatoform symptoms not explicable by organic causes were assessed with the Screening for Somatoform Symptoms scale (SOMS [12]), querying 53 physical symptoms experienced during the last seven days on a five-point Likert scale (total score 0–212). State anxiety comprising feelings of apprehension, tension, and worry was measured using the state subscale of the State-Trait Anxiety Inventory (STAI-X1, German version [13]), a 20-item instrument rated on a five-point scale (score range 0–80).

### 4.12 Pain-related cognitive-emotional mechanisms

Pain catastrophizing was measured with the Pain Catastrophizing Scale (PCS [14]), a 13-item instrument covering rumination, magnification, and helplessness, rated on a five-point scale (total score 0–52). Pain-related anxiety across cognitive, behavioral, and physiological domains was assessed with the Pain Anxiety Symptom Scale (PASS [15]), rated on a six-point scale (total score 0–240). Pain vigilance, awareness, preoccupation, and observation of pain were measured with the Pain Vigilance and Awareness Questionnaire (PVAQ [16]), a 16-item instrument rated on a six-point scale (total score 0–80). German versions of the PCS and PVAQ were used.

The overall sum score of each questionnaire was used, consistent with the exploratory character of the original assessment [2]. Scoring details and score ranges for all instruments are summarized in Table 2, and distributions of questionnaire scores grouped by neuropathic QST pattern are shown in Fig. 2.

## Limitations

QST parameters were collected only in healthy subjects, limiting generalizability to neuropathic pain patients. Two clinically relevant QST measures showed almost no variance in healthy volunteers and were therefore excluded from both the original analyses and the present dataset: paradoxical heat sensations (PHS; warmth perceived during alternating cooling and warming) and dynamic mechanical allodynia (DMA; pain evoked by normally nonpainful stroking touch). These measures are important in neuropathic pain diagnostics, as PHS indicates altered thermal perception and DMA is a marker of central sensitization, for example in complex regional pain syndrome [17]. A third parameter, vibration detection threshold (VDT), also showed low variation in healthy subjects but was retained because it still offers some discriminative value.

The QST dataset contains 18 missing values (0.74% of 2420 values). Imputation is required for many analyses, particularly machine-learning approaches, to address these gaps in pain-related data [18]. Users combining this dataset with a previously released related dataset should note partial subject overlap (n = 102; 75.6%) [5]. Psychological data include only questionnaire total scores, not subscales.

## Ethics Statement

This data set is from a previously published study that had been performed in accordance with the Declaration of Helsinki and was approved by the Ethics Committee of the Medical Faculty of the Goethe University Frankfurt am Main, Germany (reference number 27/11 and 71/12). The responsible ethics review board has explicitly approved the upload of the anonymized data to a public repository.

## CRediT Author Statement

**Jörn Lötsch:** Conceptualization, Methodology, Software, Validation, Resources, Data Curation, Writing - Original Draft, Writing - Review & Editing, Supervision, Project administration, Funding acquisition, Revision of the manuscript. **Violeta Dimova:** Project administration, Methodology, Investigation, Writing - Review & Editing.

## Data Availability

Mendeley DataIntegrated quantitative sensory testing and psychological phenotyping dataset for capsaicin-induced neuropathic pain susceptibility (Original data)
